# *De novo* assembly and comparative analysis of cherry (*Prunus* subgenus *Cerasus*) mitogenomes

**DOI:** 10.3389/fpls.2025.1568698

**Published:** 2025-03-24

**Authors:** Tianya Zhai, Zhuang Zhao, Chenlong Fu, Lizhen Huang, Changci Jiang, Meng Li, Zefu Wang, Xiaoyue Yang

**Affiliations:** ^1^ State Key Laboratory of Tree Genetics and Breeding, Co-Innovation Center for Sustainable Forestry in Southern China, College of Ecology and Environment, Nanjing Forestry University, Nanjing, China; ^2^ State Key Laboratory for Conservation and Utilization of Bio-Resource in Yunnan, School of Life Sciences, Yunnan University, Kunming, China; ^3^ College of Life Sciences, Nanjing Forestry University, Nanjing, China

**Keywords:** cherry, mitochondrial genome, comparative analysis, evolution, phylogenetic analysis

## Abstract

*Prunus* subgenus *Cerasus* (Mill) A. Gray, commonly known as cherries and cherry blossoms, possesses significant edible and ornamental value. However, the mitochondrial genomes (mitogenomes) of cherry species remain largely unexplored. Here, we successfully assembled the mitogenomes of five cherry species (*P. campanulata*, *P. fruticosa*, *P. mahaleb*, *P. pseudocerasus*, and *P.* sp*eciosa*), revealing common circular structures. The assembled mitogenomes exhibited sizes ranging from 383,398 bp to 447,498 bp, with GC content varying between 45.54% and 45.76%. A total of 62 to 69 genes were annotated, revealing variability in the copy number of protein-coding genes (PCGs) and tRNA genes. Mitogenome collinearity analysis indicated genomic rearrangements across *Prunus* species, driven by repetitive sequences, particularly dispersed repeats. Additionally, the five cherry species displayed highly conserved codon usage and RNA editing patterns, highlighting the evolutionary conservation of the mitochondrial PCGs. Phylogenetic analyses confirmed the monophyly of subg. *Cerasus*, although notable phylogenetic incongruences were observed between the mitochondrial and plastid datasets. These results provide significant genomic resources for forthcoming studies on the evolution and molecular breeding of cherry mitogenomes, enhancing the overall comprehension of mitogenome structure and evolution within *Prunus*.

## 1 Introduction

Mitochondria are crucial organelles within plant cells, primarily responsible for the synthesis of adenosine triphosphate (ATP) via the process of oxidative phosphorylation (Møller et al., 2021; Igamberdiev and Bykova, 2023). Beyond energy generation, they play critical roles in regulating cell death, maintaining calcium balance, and synthesizing various biomolecules (Green et al., 2014). Mitochondria are believed to have emerged from a symbiotic interaction between a primitive eukaryote and an ancestral prokaryote. This theory has been supported from the observed similarities between mitochondrial DNA and bacterial genomes, as well as their double-membrane structure (Sagan, 1967). Plant mitochondria possess their own genetic material, known as mitochondrial DNA, which is typically inherited maternally, although instances of paternal inheritance have been documented in certain species (McCauley, 2013; Camus et al., 2022). The slow rate of evolution in plant mitogenomes makes them valuable tools for phylogenetic studies, giving out a basic framework of the angiosperm phylogenies (Wang et al., 2024b; Hu et al., 2023a). In addition to their evolutionary significance, the mitogenomes have practical applications in plant breeding. For instance, mitochondrial mutations are linked to cytoplasmic male sterility (CMS), a trait commonly utilized in the development of hybrid crops (Zhou et al., 2024; Bohra et al., 2016).

In contrast to the single circular genomes typically found in most animal mitochondria and plant plastids, plant mitogenomes exhibit a highly complex structure with considerable variation (Yu et al., 2022; Bi et al., 2022). The structural variation observed among different species is attributed to the existence of repetitive sequences, which facilitate the dynamic rearrangement of mitochondrial DNA (Nosek and Tomáska, 2003; Wang et al., 2024b; Arimura, 2018). The size of plant mitogenomes can vary greatly, with some species showing significant genome expansion (Skippington et al., 2015; Putintseva et al., 2020). This variability is often associated with the presence of large non-coding regions, including repetitive DNA. Regarding gene content, the mitochondrial genomes of plants generally encompass both core genes that are critical for mitochondrial functionality and highly variable genes, which exhibit significant variation among different species (Sun et al., 2024; Sloan et al., 2012). These variable genes are involved in processes such as energy production and stress response, and their diversity reflects the evolutionary adaptations of plants to their respective environments (Møller et al., 2021; Van Aken et al., 2009). RNA editing events in plant mitogenomes are extensive (Stuart et al., 2005). The precise locations of RNA editing sites involve numerous cytidine-to-uridine (C-to-U) substitutions, which are predominantly influenced by *trans*-acting factors (Barkan et al., 2012); however, there are also some simple modes like direct extension (Sloan and Wu, 2016). The mutation rate of plant mitogenomes exhibits significant variability among different lineages, ranging from nearly negligible levels to as high as ten mutations per site per million years (Wang et al., 2024b, c). This variation is influenced by factors such as selection pressure (Sloan et al., 2012), mutation burden (Lynch et al., 2006), genetic drift (Lynch, 2010) and alternations in ways of inheritance (Postel et al., 2023). In addition, the copy number of plant mitogenomes is negatively correlated with both genome size and mutation rate, which are characteristics specific to mitogenomes (Zwonitzer et al., 2024).

*Prunus* subgenus *Cerasus* (Mill) A. Gray, belonging to Rosaceae (Rosales), comprises approximately 80 species and is extensively distributed across the temperate and subtropical areas of the Northern Hemisphere (Yi et al., 2024). It holds significant economic value, both in terms of edible (cherry species) and ornamental (cherry blossom species) uses. Notable edible cherry species, including *P. avium* (sweet cherry) and *P. cerasus* (sour cherry), are cultivated for their high nutritional content and commercial importance (Blando and Oomah, 2019). These fruits are rich in vitamins, minerals, and antioxidants, making them popular for fresh consumption as well as for processed products like juices, jams, and preserves (Blando and Oomah, 2019; Khadivi et al., 2024). Their unique balance of sweetness and tartness contributes to their widespread appeal in global markets. In addition to their nutritional benefits, species within subg. *Cerasus* are highly regarded for their ornamental qualities. Cherry blossoms, especially those from species like *P. serrulata*, have become iconic symbols of beauty in various cultures. These blossoms, celebrated for their fleeting yet stunning bloom, are central to numerous cultural festivals (McClellan, 2012). Ornamental cherry species symbolize renewal, fleeting beauty, and resilience, further enhancing their aesthetic value in landscape design and tourism (Kirker and Newman, 2021). Currently, studies have assembled the mitogenomes of a few cherry species like *P. avium* (Yan et al., 2019; Pervaiz et al., 2015). However, the mitogenome of subg. *Cerasus* has not been fully characterized. Critical aspects that need further investigation include the precise conformation of the mitogenome structures, intergenomic transfer (IGT) events between plastomes and mitogenomes, and a broader phylogenetic analysis with more extensive sampling.

In this study, we assembled and annotated the mitogenomes of five cherry species (*P. campanulata*, *P. fruticosa*, *P. mahaleb*, *P. pseudocerasus*, and *P.* sp*eciosa*). We compared their gene contents, sequence repeats, relative synonymous codon usage (RSCU), RNA editing sites, and mitochondrial plastid sequences (MIPTs). Additionally, we analyzed genome synteny and phylogenetic relationships with other closely related species. The results yield valuable insights into the characteristics and evolution of subg. *Cerasus* mitogenomes, as well as significant data for the phylogeny and genetic information of cherry species.

## 2 Materials and methods

### 2.1 Plant sampling and DNA sequencing

Fresh leaves of *P. mahaleb* were collected from the Shanghai Chenshan Botanical Garden (Shanghai, China; 31°4’ N, 121°10’ E) and stored at -80°C. DNA was extracted using a modified Cetyltrimethylammonium Bromide (CTAB) method. The purity and quality of the DNA samples were assessed utilizing AMPure PB magnetic beads and the Qubit 2.0 fluorometer (Thermo Fisher Scientific, USA). Sequencing libraries were constructed with the SMRTbell Express Template Prep Kit 3.0 (PacBio Biosciences, Menlo Park, CA, USA), and HiFi sequencing data were generated using the PacBio Revio platform. We utilized Fastp (Chen et al., 2018) for the quality control of sequencing data. Additionally, published HiFi sequencing data for three cherry species were downloaded from the National Genomics Data Center (NGDC; https://ngdc.cncb.ac.cn/): *P. campanulata* (PRJNA895162) (Hu et al., 2023b), *P. fruticosa* (PRJNA922242) (Goeckeritz et al., 2023), and *P. pseudocerasus* (PRJCA010538) (Jiu et al., 2024). HiFi reads for *P.* sp*eciosa* were obtained from NCBI (https://www.ncbi.nlm.nih.gov/) under BioProject PRJDB17512.

### 2.2 Assembly and annotation of mitogenomes

The HiFi sequencing data of five cherry species were processed using PMAT v2.0.1 (https://github.com/aiPGAB/PMAT2) to assemble their mitogenomes with the ‘autoMito’ mode (Bi et al., 2024). The assembly was performed with the parameters ‘-t hifi -m’, and the ‘-g’ parameter was set based on the nuclear genome size, as suggested by Hu et al. (2023), Goeckeritz et al. (2023); Jiu et al. (2024), and Fujiwara et al. (2024). The seed contigs were selected based on core mitochondrial genes in PMAT and coverage depth (**Supplementary Table S1**). Raw assembly graphs of the mitogenomes were visualized and manually refined using Bandage v0.9.0 (Wick et al., 2015). The online tool PMGA (http://www.1kmpg.cn/ipmga/) (Li et al., 2024) was then used to annotate the PCGs, tRNA genes, and rRNA genes with default parameters. Final annotations were reviewed and manually corrected using Geneious Prime 2023 (Kearse et al., 2012). Circular mitogenome maps were generated and visualized with OGDRAW v1.3.1 (Greiner et al., 2019).

### 2.3 Analysis of repeated sequences

Simple sequence repeats (SSRs) were detected utilizing the online tool MISA (https://webblast.ipk-gatersle-ben.de/misa/) (Beier et al., 2017). The established minimum repeat thresholds for monomeric, dimeric, trimeric, tetrameric, pentameric, and hexameric SSRs were determined to be 10, 5, 4, 3, 3, and 3, respectively. The identification of tandem repeats was conducted utilizing Tandem Repeats Finder v4.09.1 (Benson, 1999) with default settings. Dispersed repeats within the mitogenomes were detected utilizing the online resource REPuter (https://bibiserv.cebitec.unibielefeld.de/reputer/) (Kurtz et al., 2001), applying a Hamming distance of 3, a maximum of 5000 computed repeats, and a minimum repeat size of 30.

### 2.4 Analysis of codon usage and RNA editing

The PCGs of the mitogenomes were extracted utilizing PhyloSuite v1.2.3 (Zhang et al., 2020; Xiang et al., 2023). The RSCU for each mitogenome was analyzed using DAMBE7 (Xia, 2018). RNA editing sites in the mitogenomes were predicted utilizing the Predictive RNA Editor for Plants (PREP) suite (http://prep.unl.edu/) (Mower, 2009).

### 2.5 DNA transfer analysis between mitogenomes and plastomes

We assembled the plastomes of the five cherry species using PMAT v2.0.1 (Bi et al., 2024) based on the HiFi sequencing data and manually refined the results using Geneious Prime 2023 (Kearse et al., 2012). The plastid genomes were then annotated utilizing PGA (Qu et al., 2019). To identify fragments transferred from plastomes to mitogenomes, we used BLASTn v2.14.0 (Chen et al., 2015) on NCBI. The criteria for screening encompassed a matching rate that exceeded 70% and a sequence length that was greater than 50 bp. BLASTn was run with the following parameters: ‘-evalue 1e-5 -word_size 9 -gapextend 2 -reward 2 -penalty -3 -gapopen 5’.

### 2.6 Synteny and phylogenetic analysis

We compared the mitogenomes of 15 *Prunus* species, including five newly assembled in this study and ten previously published ones (**Supplementary Table S10**), using minimap2 v2.1 with the -asm5 parameter (Li, 2018). NGenomeSyn (He et al., 2023) was employed to filter collinear fragments with a minimum alignment length of 40 bp, and it also served as a tool for visualizing the synteny outcomes.

For phylogenetic analysis, we used mitochondrial and plastid genomes of 36 species, including five newly assembled in this study and 31 previously published ones downloaded from NCBI. We selected 26 species from 11 genera within Rosaceae, along with nine species from five related families (Cannabaceae, Elaeagnaceae, Moraceae, Rhamnaceae, and Ulmaceae) as ingroups. *Leucaena trichandra* from Fabaceae was used as the outgroup. In the genus *Prunus*, nine species from subg. *Cerasus* and six species from subg. *Prunus* were sampled. A total of 35 mitochondrial and 75 plastid conserved PCGs were extracted using PhyloSuite v1.2.3 (Zhang et al., 2020; Xiang et al., 2023). Sequences were aligned using MAFFT v7.407 (Katoh and Standley, 2013), and the mitochondrial and plastid alignments were concatenated separately using PhyloSuite. The concatenated alignment matrix was trimmed with the software trimAL v1.5 (Capella-Gutiérrez et al., 2009) using the ‘-automated1’ parameter. The phylogenetic tree was constructed utilizing the maximum likelihood (ML) approach as executed in RAxML-NG2 v1.2.0 (Kozlov et al., 2019). The model ‘GTR+FO+I+G4’ was identified as the most suitable option for the construction of ML tree. A bootstrap analysis was conducted with 1000 replicates to assess the robustness of the tree. Subsequently, the ML tree was visualized utilizing FigTree v1.4.4 (http://tree.bio.ed.ac.uk/software/figtree/).

## 3 Results

### 3.1 Mitogenome features of the five cherry species

**Figure 1:**
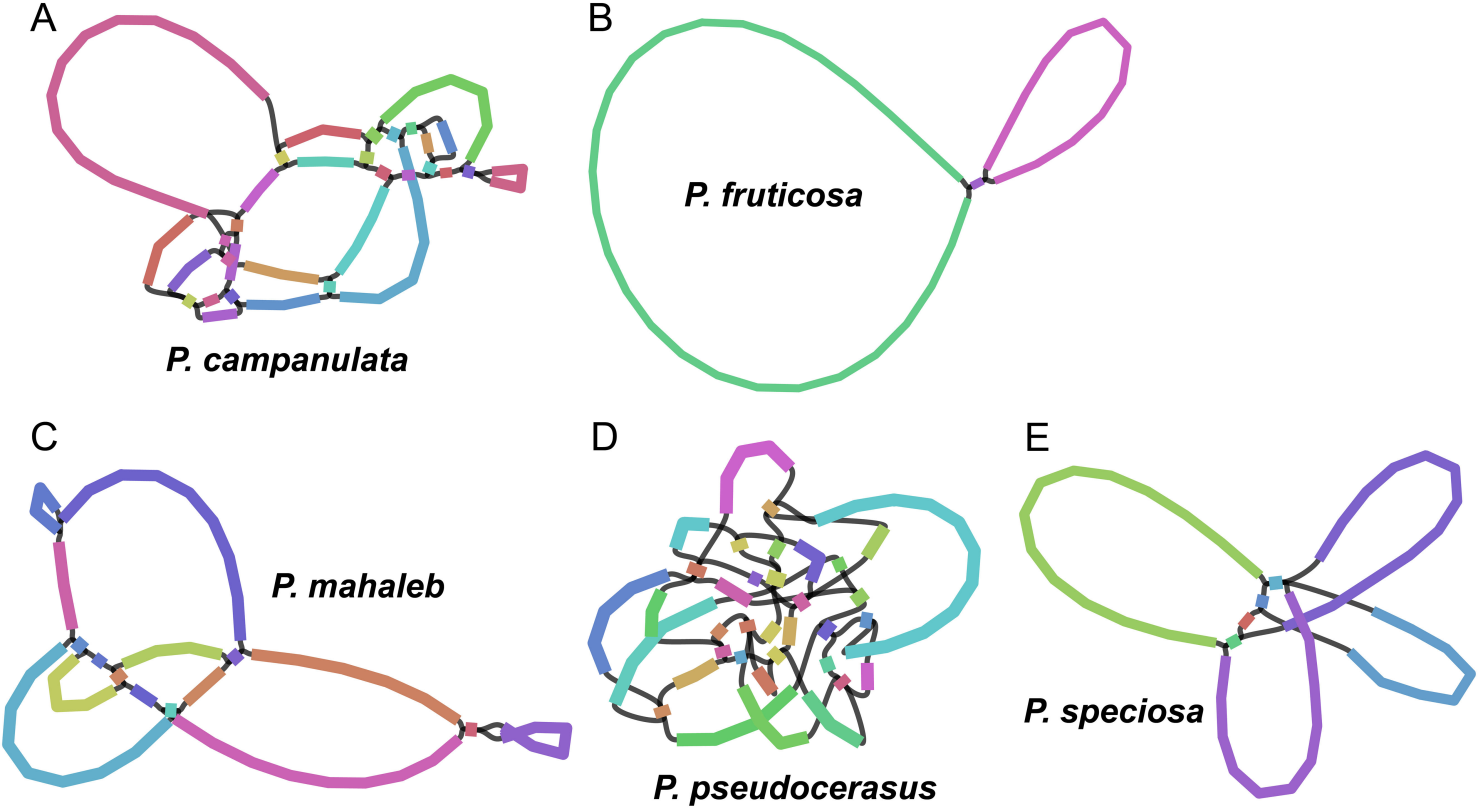
Mitogenome structure of five cherry mitogenomes. **(A)** *P. campanulate*. **(B)** *P. fruticosa*. **(C)** *P. mahaleb*. **(D)** *P. pseudocerasus*. **(E)** *P.* sp*eciosa*.

**Figure 2:**
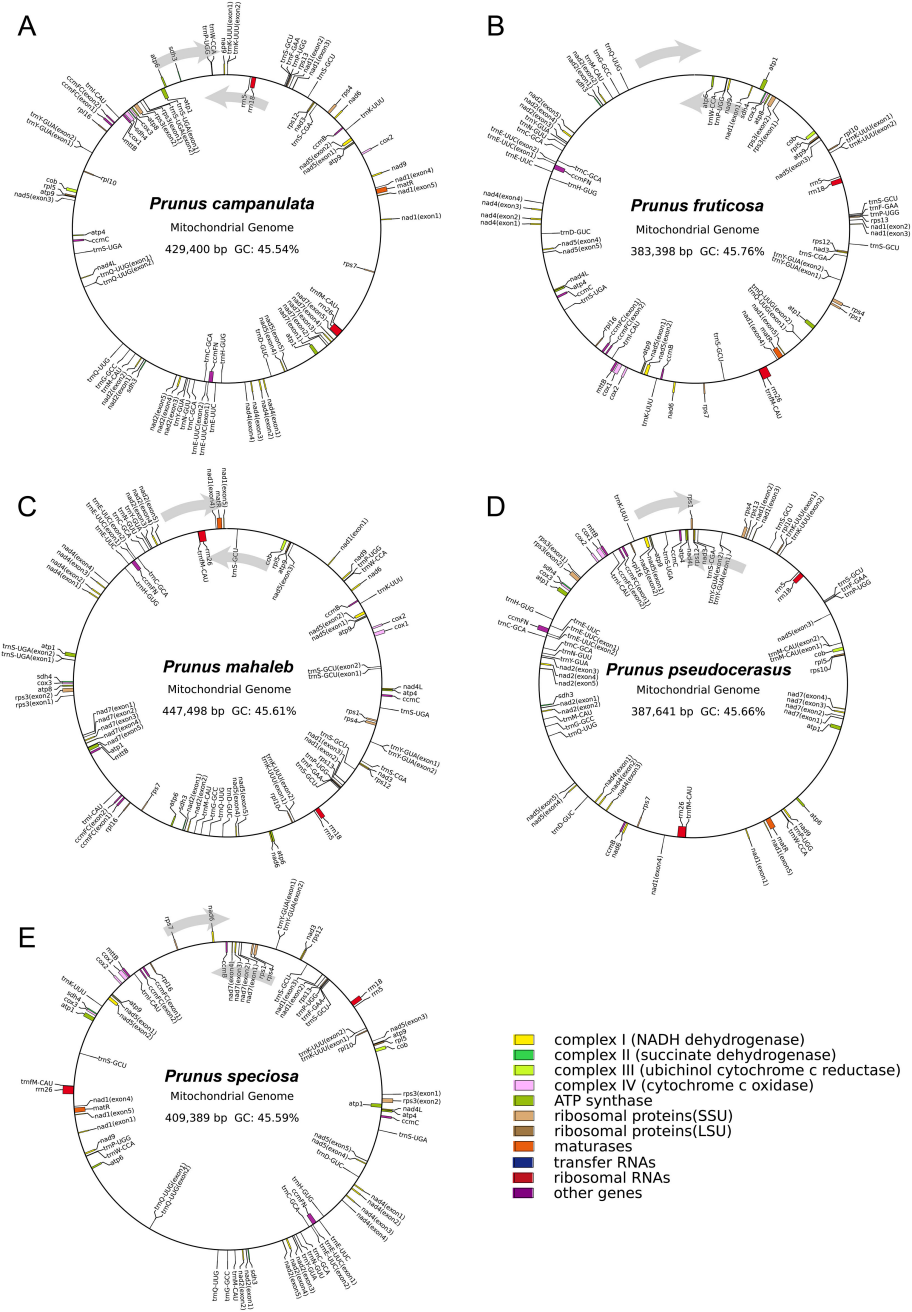
Mitogenome maps of five cherry mitogenomes. **(A)** *P. campanulate*. **(B)** *P. fruticosa*. **(C)** *P. mahaleb*. **(D)** *P. pseudocerasus*. **(E)** *P.* sp*eciosa*. Genes shown on the outside and inside of the circle are transcribed clockwise and counterclockwise, respectively. Genes belonging to different functional groups are color-coded.

We assembled mitogenomes of *P. campanulata*, *P. fruticosa*, *P. mahaleb*, *P. pseudocerasus*, and *P.* sp*eciosa* based on 27.50 Gb, 48.20 Gb, 29.47 Gb, 42.48 Gb and 35.6 Gb HiFi data, respectively. Visualization of mitogenome structures revealed that the mitogenome of *P. fruticosa* consists of three main contigs, while the mitogenomes of *P. campanulata*, *P. mahaleb*, *P. pseudocerasus*, and *P.* sp*eciosa* exhibit more complex structures with 33, 17, 36 and 8 contigs, respectively (**Figure 1**; **Supplementary Table S1**). Following the removal of duplicated regions, we identified a single putative circular chromosome for each of the five cherry mitogenomes (**Figure 2**). The mitogenome sizes of the five cherry species were determined as follows: 429,400 bp for *P. campanulata*, 383,398 bp for *P. fruticosa*, 447,498 bp for *P. mahaleb*, 387,641 bp for *P. pseudocerasus*, and 409,389 bp for *P.* sp*eciosa* (**Table 1**). The overall GC contents were found to be 45.54%, 45.76%, 45.61%, 45.66% and 45.59% for *P. campanulata*, *P. fruticosa*, *P. mahaleb*, *P. pseudocerasus*, and *P.* sp*eciosa*, respectively (**Table 1**).

**Table 1:** Basic information of five cherry mitogenomes.

Species	Length (bp)	GC Content (%)	Number of Genes	Number of PCGs	Number of tRNAs	Number of rRNAs
*P. campanulata*	429,400	45.54	66	37	26	3
*P. fruticosa*	383,398	45.76	65	36	26	3
*P. mahaleb*	447,498	45.61	69	39	27	3
*P. pseudocerasus*	387,641	45.66	62	34	25	3
*P.* sp*eciosa*	409,389	45.59	64	36	25	3

**Figure 3:**
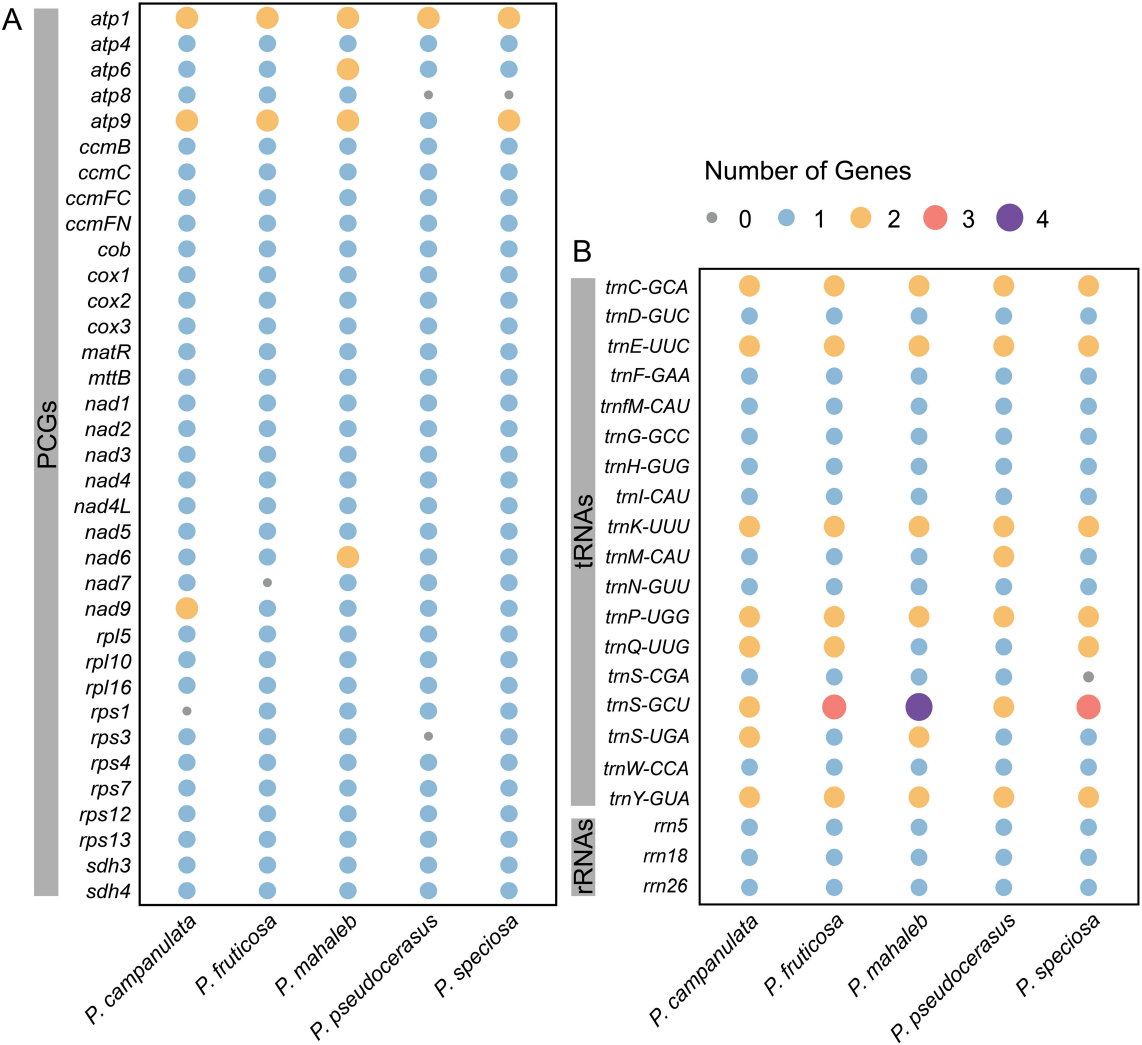
Gene content of five cherry mitogenomes. **(A)** The PCG content of five cherry mitogenomes. **(B)** The tRNA and rRNA content of five cherry mitogenomes. Different colors and sizes represent the number of genes present in each genome.

A total of 66, 65, 69, 62 and 64 genes were annotated in the mitogenomes of *P. campanulata*, *P. fruticosa*, *P. mahaleb*, *P. pseudocerasus*, and *P.* sp*eciosa*, respectively (**Table 1**). The mitogenomes of *P. campanulata*, *P. fruticosa*, *P. mahaleb*, *P. pseudocerasus* and *P.* sp*eciosa* contain 37, 36, 39, 34 and 36 PCGs, as well as 26, 26, 27, 25 and 25 tRNA genes (**Table 1**). Each of the five mitogenomes has three rRNAs genes (**Table 1**). We annotated a total of 24 core genes and 11 variable genes across all PCGs (**Figure 3**; **Supplementary Table S2**). Notably, six of the core genes (*atp6*, *atp8*, *atp9*, *nad6*, *nad7* and *nad9*) and two of the variable genes (*rps1* and *rps3*) exhibit quantitative variations among the five mitogenomes (**Figure 3A**; **Supplementary Table S2**). Additionally, five tRNA genes (*trnM-CAU*, *trnQ-UUG*, *trnS-CGA*, *trnS-GCU* and *trnS-UGA*) displayed copy number variations across the five mitogenomes, whereas all rRNA genes (*rrn18*, *rrn5* and *rrn26*) were found to be consistent in number across each of the five mitogenomes (**Figure 3B**; **Supplementary Table S2**). These findings indicated that the gene content of the five mitogenomes is relatively conserved, while variations in the number of genes are present.

### 3.2 Repeats analysis of the five cherry mitogenomes

**Figure 4:**
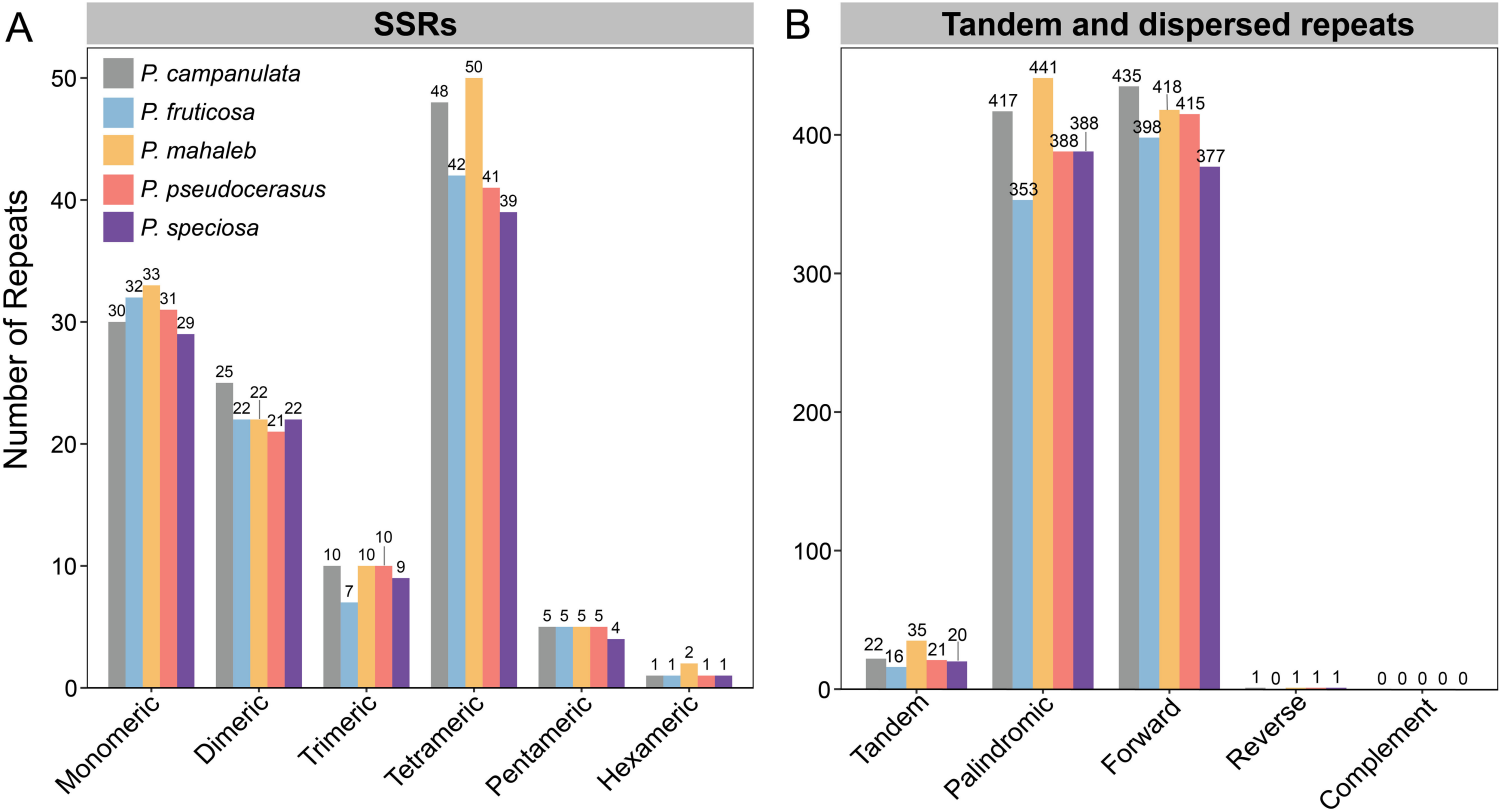
Detected repeats in five cherry mitogenomes. **(A)** Type and number of detected SSRs. **(B)** Type and number of detected tandem and dispersed repeats.

The dynamic recombination of mitogenomes is primarily driven by the presence of repeats. Here, we conducted predictions of SSRs, tandem repeats, and dispersed repeats within the mitogenomes of five cherry species. A total of 118, 109, 122, 109, and 104 SSRs were identified in the mitogenomes of *P. campanulata*, *P. fruticosa*, *P. mahaleb*, *P. pseudocerasus*, and *P.* sp*eciosa*, respectively (**Figure 4A**; **Supplementary Table S3**). Tetrameric SSRs were the most common, accounting for 37.50% to 40.98% of the total SSRs across the five mitogenomes (**Figure 4A**; **Supplementary Table S3**). Conversely, hexameric SSRs were the least common, accounting for only 0.0084% to 0.016% of total SSRs across the five mitogenomes (**Figure 4A**; **Supplementary Table S3**). Among the monomeric SSRs, adenine (A) monomer repeats dominated in *P. campanulata*, *P. fruticosa*, and *P.* sp*eciosa*, while thymine (T) monomers were most prevalent in *P. mahaleb* and *P. pseudocerasus* (**Supplementary Table S3**). These results indicate that the overall characteristics of SSRs across the five mitogenomes are similar, with differences only in the specific nucleotide compositions.

We detected 22, 16, 35, 21, and 20 tandem repeats in the mitogenomes of *P. campanulata*, *P. fruticosa*, *P. mahaleb*, *P. pseudocerasus*, and *P.* sp*eciosa*, respectively (**Figure 4B**; **Supplementary Table S4**). In addition, 853, 751, 860, 804, and 766 dispersed repeats were also identified in the mitogenomes of *P. campanulata*, *P. fruticosa*, *P. mahaleb*, *P. pseudocerasus*, and *P.* sp*eciosa*, respectively (**Figure 4B**; **Supplementary Table S5**). Among the dispersed repeats, palindromic repeats account for the highest proportion in *P. mahaleb* and *P.* sp*eciosa*, while forward repeats account for the highest proportion in *P. campanulata*, *P. fruticosa* and *P. pseudocerasus* (**Figure 4B**; **Supplementary Table S5**). No complement repeats were detected within the five mitogenomes. The results further showed that the patterns of the five mitogenomes repeats exhibit minor variations yet remain fundamentally consistent.

### 3.3 Codon usage analysis and prediction of RNA editing sites of the PCGs

**Figure 5:**
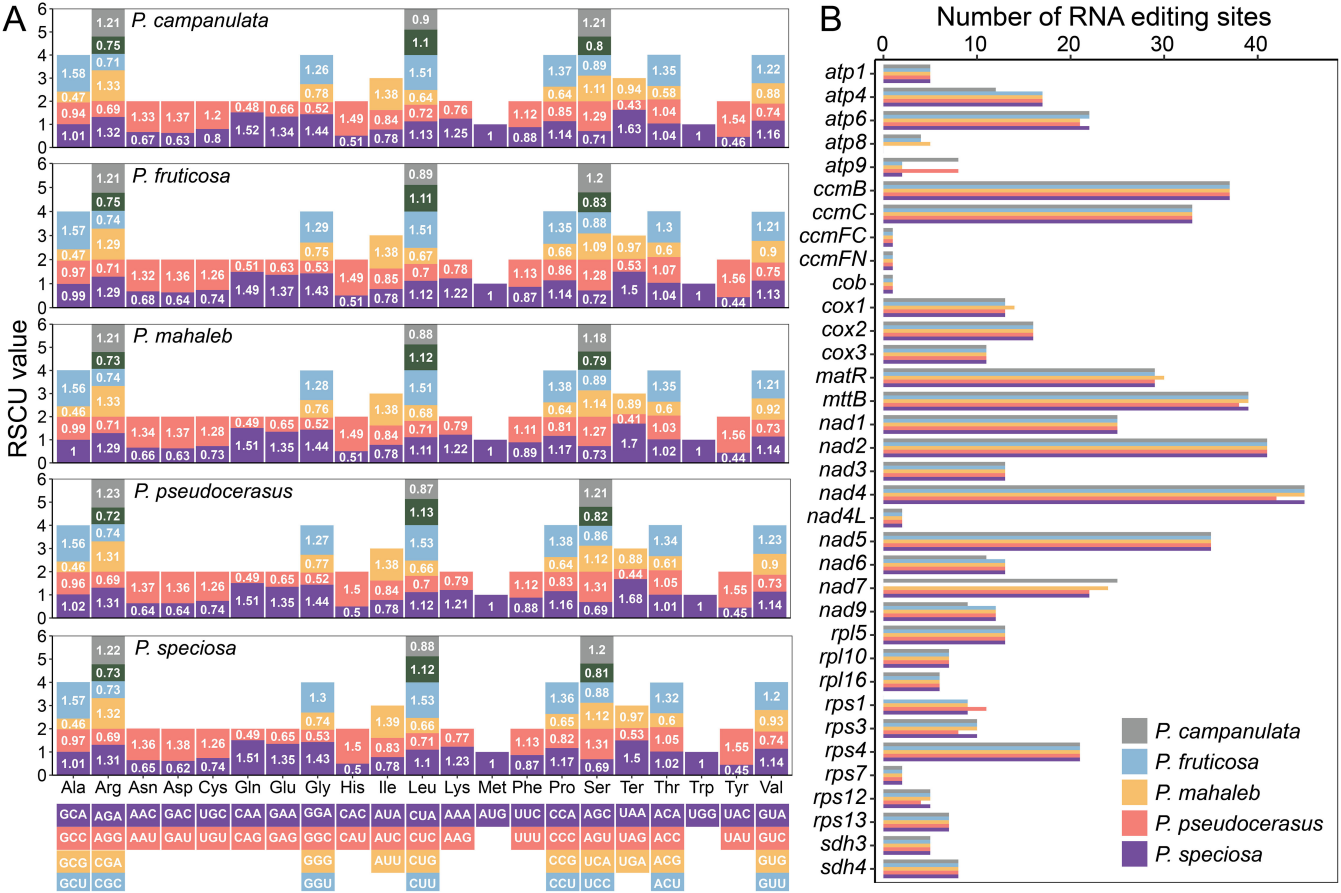
Analysis of RSCU in five cherry mitogenomes and number of RNA editing sites identified in each PCG of five cherry mitogenomes. **(A)** The X-axis displays various codon families and the RSCU values reveal the frequency of specific codons on usage. **(B)** The X-axis represents the number of RNA editing sites, and the Y-axis represents protein coding genes.

A total of 10,601 (*P. campanulata*), 10,376 (*P. fruticosa*), 11,141 (*P. mahaleb*), 10,408 (*P. pseudocerasus*), and 10,534 (*P.* sp*eciosa*) codons were analyzed to explore codon usage patterns (**Supplementary Table S6**). Across the five mitogenomes, the most frequent amino acids were arginine (Arg), leucine (Leu), and serine (Ser). The secondary common amino acids included alanine (Ala), glycine (Gly), proline (Pro), threonine (Thr), and valine (Val), while methionine (Met) and tryptophan (Trp) are relatively rare (**Figure 5A**). The overall RSCU values of the five mitogenomes were similar. RSCU values greater than 1 indicate an amino acid preference, and values for the start codons AUG and UGG are exactly 1 across all mitogenomes (**Figure 5A**; **Supplementary Table S6**). For *P. campanulate*, *P. mahaleb* and *P. pseudocerasus*, UAA (Ter) exhibited the highest RSCU values, while for *P. fruticosa* and *P.* sp*eciosa*, GCU (Ala) had the highest RSCU values (**Figure 5A**; **Supplementary Table S6**). The observation indicated that the five mitogenomes are basically same in Codon Usage, with only partial differences on the maximum value.

We annotated 522, 510, 536, 528, and 528 RNA editing sites in the mitogenomes of *P. campanulata*, *P. fruticosa*, *P. mahaleb*, *P. pseudocerasus*, and *P.* sp*eciosa*, respectively (**Figure 5B**; **Supplementary Table S7**). Among the mitochondrial genes analyzed, *nad4* demonstrated the highest number of editing sites across all mitochondrial genes for the five mitogenomes (**Figure 5B**; **Supplementary Table S7**). In contrast, *ccmFC*, *ccmFN* and *cob* were predicted to have the lowest number of editing sites, with only one site for all the five mitogenomes (**Figure 5B**; **Supplementary Table S7**). Notably, without considering the missing genes, the types and numbers of RNA editing sites in the *P. fruticosa* are exactly the same as those in *P.* sp*eciosa*. The results showed the high similarity in RNA editing events across the five mitogenomes.

### 3.4 Analysis of mitochondrial plastid sequences

**Figure 6:**
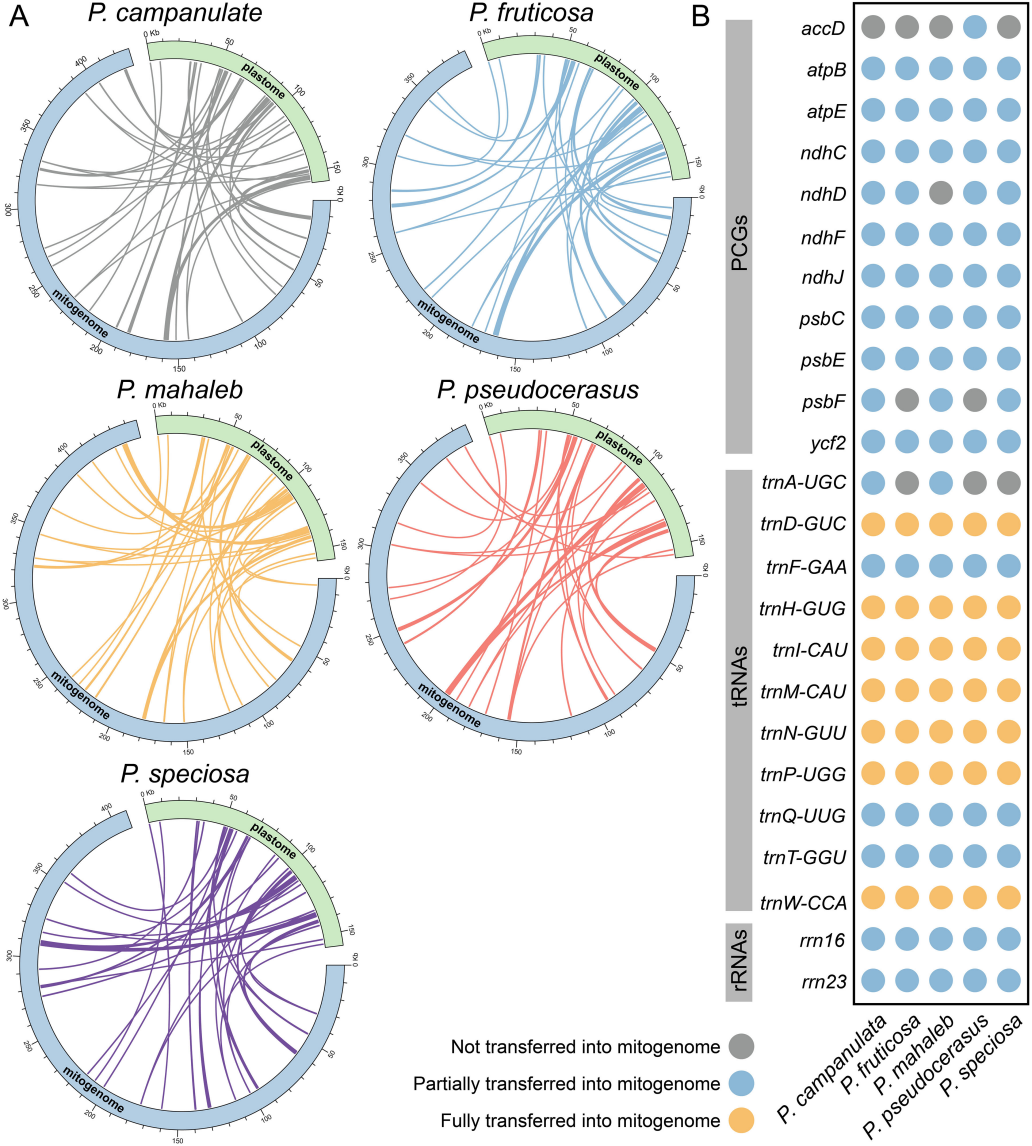
The DNA fragments transfer that occurred between mitogenomes and plastomes of five cherry species. **(A)** MIPTs on the mitogenomes and plastomes. The blue and green sections represent the mitogenomes and the plastomes, respectively. **(B)** The 24 annotated plastid genes of MIPTs in the five cherries. The gray, blue and yellow dots represent plastid genes that not partially and fully transferred into mitogenomes, respectively.

The models of MIPTs in the five cherry species are presented in **Figure 6A**. We identified 54, 50, 54, 47, and 50 MIPTs in the mitogenomes of *P. campanulata*, *P. fruticosa*, *P. mahaleb*, *P. pseudocerasus*, and *P.* sp*eciosa*, respectively, using a similarity threshold of 70% (**Supplementary Table S8**). The longest MIPT fragment spanned 1,603 bp (**Supplementary Table S8**). A total of 24 plastid genes were annotated in these homologous fragments (**Figure 6B**; **Supplementary Table S9**). However, only seven of these genes were fully transferred into mtDNA, and all fully transferred genes are tRNAs (**Figure 6B**; **Supplementary Table S9**). Among the transferred genes, *trnA*-*UGC*, *psbF*, *ndhD*, and *accD* exhibited varying transfer statuses across the five mitogenomes, with transfer occurring in only some of the species (**Figure 6B**; **Supplementary Table S9**). These findings indicated that the MIPTs patterns across the five mitogenomes are largely consistent, with minor differences in the transfer of specific fragments and genes.

### 3.5 Synteny and phylogenetic analysis

**Figure 7:**
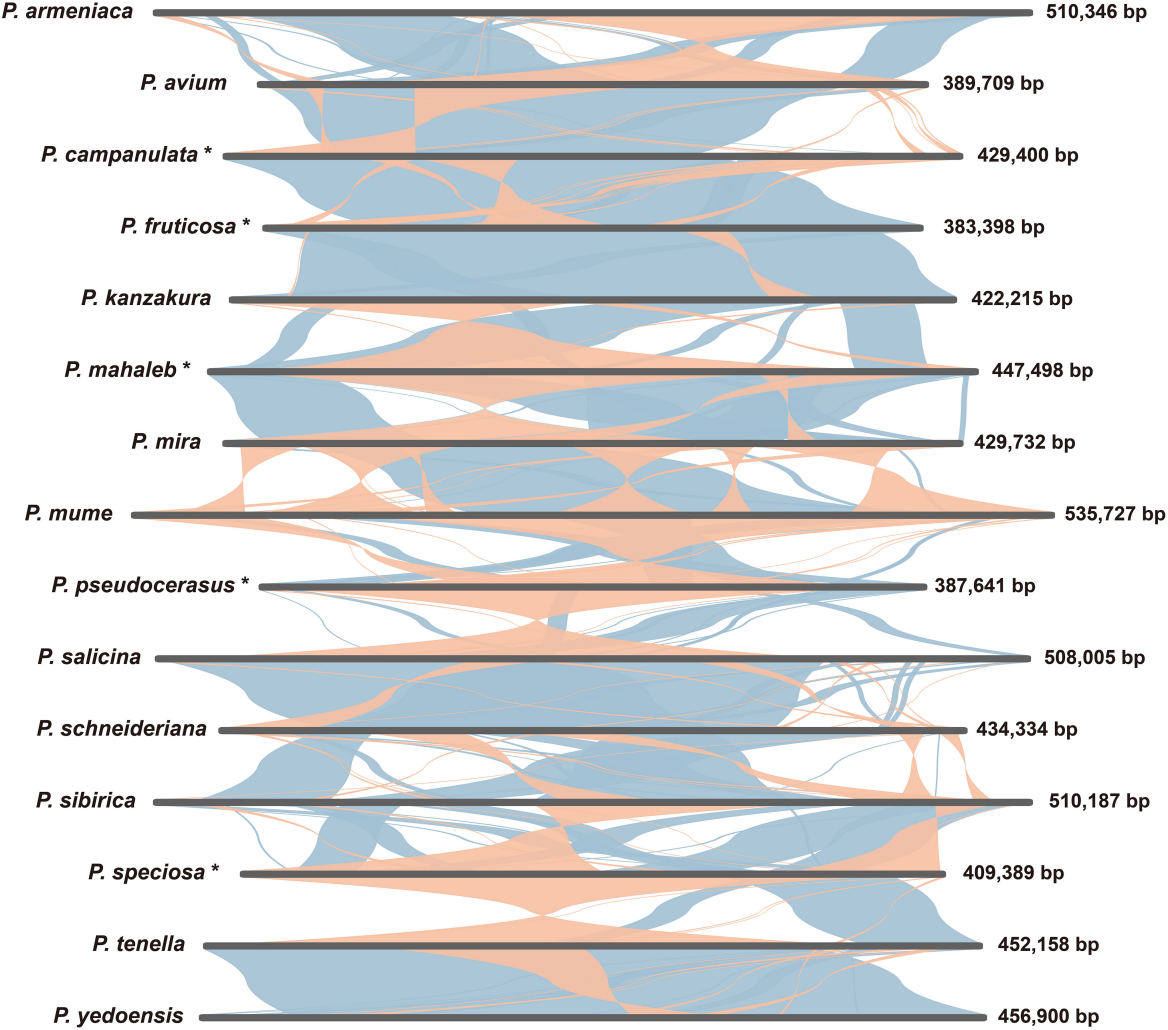
Mitogenome synteny analysis of 15 *Prunus* species. The bars indicate the mitogenomes of the species, the blue ribbons represent forward homologous sequences between the species, and the orange ribbons represent reverse homologous sequences between the species.

We undertook a comparative analysis to examine the collinear regions present in the mitogenomes of the five cherry species and other ten *Prunus* species (**Figure 7**). We totally identified 223 collinear fragments, with a total size of 7,302,323 bp. There is an average of 17 collinear fragments present between the mitogenomes of each pair of species (**Supplementary Table S10**). The largest collinear fragment, spanning 361,941 bp, was observed between *P.* sp*eciosa* and *P. tenella*, whereas the shortest collinear fragment was found between *P. tenella* and *P. yedoensis*, with a length of 257 bp (**Supplementary Table S9**). The findings indicated significant variations in the configuration and alignment of various collinear fragments within the mitochondrial genomes of the examined species (**Figure 7**). This evidence implied that the mitochondrial genomes of these 15 *Prunus* species have experienced considerable rearrangement.

**Figure 8:**
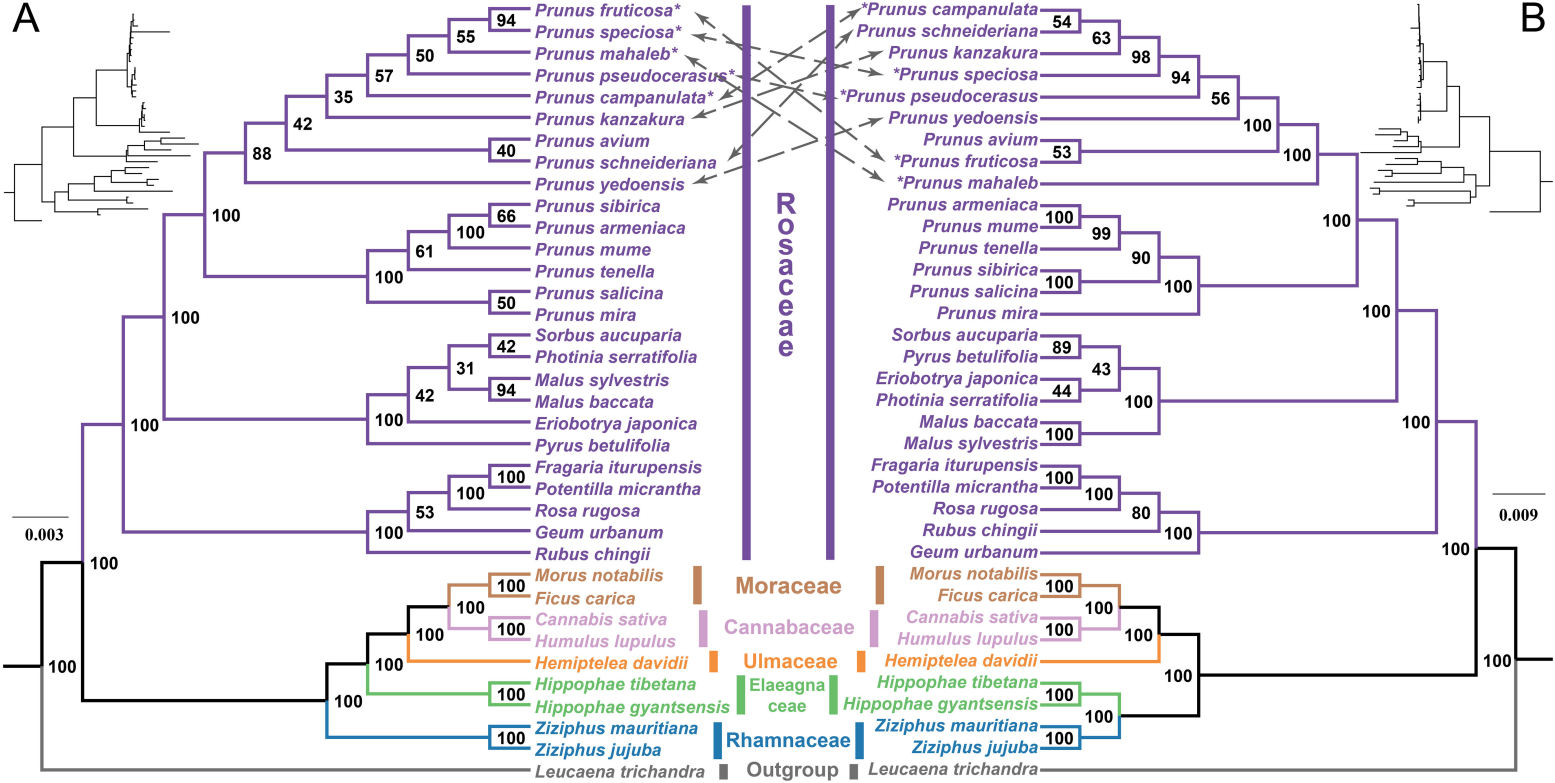
Phylogenetic relationships of five cherry species and other closely related species based on mitochondrial and plastid datasets. **(A)** Maximum likelihood phylogenetic tree based on 35 PCGs of mitogenomes. The numbers on the branch represent the bootstrap values. **(B)** Maximum likelihood phylogenetic tree based on 75 PCGs of plastomes. The number on the branch represents the bootstrap values. The gray dashed lines point to cherry species that occupy different positions in the two phylogenetic trees. The species marked with an asterisk (*) are the five new cherry species included in this study.

We constructed the phylogenetic trees based on 35 mitochondrial PCGs and 75 plastid PCGs from five families (Rosaceae, Moraceae, Cannabaceae, Ulmaceae, Elaeagnaceae, and Rhamnaceae) to ascertain the evolutionary status of the five cherry species (**Supplementary Table S11**). At the family level, the phylogenetic tree constructed by mitogenomes was consistent with that of plastid tree, exhibiting 100% bootstrap support (**Figure 8**). This result supported the latest classification established by the Angiosperm Phylogenetic Group (APG IV) (The Angiosperm Phylogeny Group, 2016). In the family Rosaceae, the phylogenetic relationships of some genera in the mitochondrial tree do not agree with those inferred from the plastid tree (**Figure 8**). However, the division between *Prunus* subg. *Cerasus* and *Prunus* subg. *Prunus* was supported with 100% bootstrap value in both trees (**Figure 8**). Within the subg. *Cerasus*, the phylogenetic relationships among species of the mitochondrial tree do not consistently match those derived from the plastid tree, and all the bootstrap values are less than 100% (**Figure 8**). In the mitochondrial tree, the topological relationship among the five cherry species was represented as (*P. campanulata*, (*P. pseudocerasus*, (*P. mahaleb*, (*P.* sp*eciosa*, *P. fruticosa*)))), in contrast, the topology was (*P. mahaleb*, (*P. fruticosa*, (*P. pseudocerasus*, (*P.* sp*eciosa*, *P. campanulata*)))) in the plastid tree.

## 4 Discussion

### 4.1 Complex structures of cherry mitogenomes

Early studies believed that circular genome-sized molecules constituted the predominant form of mitochondrial DNA in plants (Palmer and Shields, 1984). However, the structures of plant mitogenomes are, in fact, more complex. Fluorescence microscopy analyses have revealed that plant mitochondrial DNA contains linear, branched, and circular structures (Kozik et al., 2019), and this structural complexity is typically ascribed to recombination processes facilitated by the presence of numerous repetitive sequences (Wang et al., 2024b). Mitogenome structures have been characterized in a variety of species, revealing a wide range of configurations across plants. For example, the mitogenomes of *Ilex rotunda* (Wang et al., 2024d) and *Salix purpurea* (Han et al., 2022) consisted of a single circular molecule, while *Lycopodium japonicum* (Sun et al., 2024) had a mitogenome composed of three contigs. The *Populus simonii* (Bi et al., 2022) mitogenome was assembled into three circular molecules, and *Cinnamomum camphora* (Han et al., 2024) exhibited a more intricate branched structure. In this study, we visualized the assembled mitogenomes of five cherry species, uncovering typical circular structures of varying complexity (**Figure 1**). Similar circular structures have also been observed in other *Prunus* species, such as *P. salicina* and *P. salicina* (Fang et al., 2021; Liu et al., 2024). The mitogenome of *P. fruticosa* displayed a structure comprising three contigs, whereas the mitogenome of *P. pseudocerasus*, exhibiting the most complex structure, contains 36 contigs. The complex mitogenome structures in cherry species align with the current understanding of plant mitogenomes, suggesting that circular structures may be prevalent among cherry species.

### 4.2 Variations in mitogenome size and gene content

Plant mitochondrial PCGs evolve at a slower rate compared to nuclear and plastid genomes (Wolfe et al., 1987). However, there exists significant variability in both the size of genomes and the composition of genes. Studies have shown that plant mitogenome sizes range from 66 Kb (*Viscum scurruloideum*) (Skippington et al., 2015) to 11.7 Mb (*Larix sibirica*) (Putintseva et al., 2020). The PCGs of land plant mitogenomes encompass both core and variable genes. Despite the conserved nature of core genes, gene loss and gain have occurred across different lineages (Mower, 2020), leading to variation in gene content. Similarly, the number of tRNA genes exhibits varying levels of variability (Bi et al., 2022). Previous studies have assembled the mitogenomes of several *Prunus* species, including *P. avium* (389,709 bp) (Yan et al., 2019), *P. salicina* (508,035 bp) (Fang et al., 2021), and *P. tenella* (452,158 bp) (Liu et al., 2024). In this study, the mitogenomes of five cherry species were newly assembled, with sizes ranging from 383,398 bp (*P. fruticosa*) to 447,498 bp (*P. mahaleb*) (**Table 1**), extending the range of mitogenome size variation within *Prunus*. Our results indicated that there are no differences in the content or copy number of rRNA genes across the five cherry mitogenomes, which is consistent with other *Prunus* species (Yan et al., 2019; Fang et al., 2021; Liu et al., 2024). Among the PCGs, *atp8*, *nad7*, *rps1*, and *rps2* genes were lost in certain species, while *atp6*, *atp9*, *nad6*, and *nad9* genes exhibited copy number variation (**Figure 3**). For tRNA genes, the *trnS*-*CGA* gene is absent in *P.* sp*eciosa*, while the *trnM*-*CAU*, *trnQ*-*UUG*, *trnS*-*GCU*, and *trnS*-*UGA* genes display copy number variations (**Figure 3**). We noted that the loss of *rps2* is also found in *Prunus salicina* and *Prunus salicina*, and that *Prunus salicina* has also lost *trnS-CGA* (Fang et al., 2021; Liu et al., 2024). These results enhanced the comprehension of the variability in mitogenome size and gene content within *Prunus*.

### 4.3 MIPT sequences, repetitive elements, and genome rearrangements

Studies of organelle genome have indicated that most plants have experienced unidirectional fragment migration from the plastome to the mitogenome, with only a few exceptions where fragments have been transferred in the opposite direction (Notsu et al., 2002; Song et al., 2023; Lian et al., 2024). In our study, MIPTs were identified and were found to be similarly abundant in cherry species (**Figure 6**; **Supplementary Table S8**). Notably, all seven fully transferred genes are tRNAs, which lends support to the hypothesis that the transfer of tRNA genes from the plastid genome to the mitochondrial genome is a prevalent phenomenon among angiosperms (Timmis et al., 2004). Interestingly, these seven tRNAs were also found to be completely transferred in *Prunus tenella* (Liu et al., 2024), suggesting conservation among *Prunus* species. Repetitive sequences are prevalent in plant mitogenomes, and recombination mediated by dispersed repetitive sequences has been demonstrated in numerous studies (Wang et al., 2024a; Li et al., 2021; Yu et al., 2022). In this study, we identified SSRs, tandem repeats, and dispersed repeats within the mitogenomes of five cherry species. Among these, the length of dispersed repeats varied between 34,110 bp and 41,088 bp across the five mitogenomes, suggesting their potential role in mitogenome recombination and structural complexity (**Figure 4B**; **Supplementary Table S4**). The proportion of dispersed repeated sequences in the five mitogenomes ranges from 8.33% to 9.57% (**Figure 4B**; **Supplementary Table S4**). This is comparable to *Prunus tenella*, which has a proportion of 10.39% (Liu et al., 2024), lower than that of *Lycopodium japonicum* (27.42%) (Sun et al., 2024), and higher than the 2.35% observed in *Populus deltoides* (Qu et al., 2023). The collinearity analysis of 15 *Prunus* mitogenomes revealed extensive genomic rearrangements (**Figure 7**; **Supplementary Table S10**). The large number of repetitive sequences, particularly dispersed repeats, are closely linked to the significant rearrangements observed in the mitogenomes of cherry species.

### 4.4 Similarities of codon usage patterns and RNA editing sites

RSCU analysis is important as it provides insights into the evolutionary patterns of codon usage, which can reflect selective pressures on mitochondrial genes and their functional constraints (Parvathy et al., 2022). The analysis of the RSCU in mitochondrial PCGs revealed that the mitogenomes of five cherry species exhibit highly similar codon usage biases (**Figure 5A**; **Supplementary Table S6**). RNA editing is a widespread phenomenon observed in plant mitogenomes, and it significantly contributes to protein folding by affecting post-transcriptional gene expression and modifying the genetic information present in mRNA (Small et al., 2020; Lu, 2018). Studies have demonstrated considerable variation in the number of RNA editing sites across different plant species’ mitogenomes. For instance, *Arabidopsis thaliana* contains 441 RNA editing sites in 36 PCGs (Unseld et al., 1997), *Suaeda glauca* has 216 RNA editing sites in 26 PCGs (Cheng et al., 2021), and *Oryza sativa* exhibits 491 RNA editing sites in 34 PCGs (Notsu et al., 2002). In this study, we predicted 510–536 RNA editing sites across the five mitogenomes (**Figure 5B**; **Supplementary Table S7**). Despite variations in the number of RNA editing sites, the overall trends across the five cherry species are highly similar. These findings provide further evidence of the conservation of mitochondrial PCGs in cherry species.

### 4.5 Phylogenetic incongruences between mitochondrial and plastid datasets

Plant mitochondrial PCGs evolve at a slower rate, making them crucial for phylogenetic studies, particularly those involving ancient evolutionary lineages and clades with faster evolutionary rates (e.g., parasitic lineages (Qiu et al., 2010; Hu et al., 2023a)). In this study, we reconstructed the phylogenetic relationships of 36 angiosperm species based on mitochondrial PCGs, including nine subg. *Cerasus* species and six subg. *Prunus* species. The results revealed that *Prunus* splits into two clades with 100% bootstrap support: subg. *Cerasus* and subg. *Prunus*. Within subg. *Cerasus*, the interspecific relationships were moderately supported, with bootstrap values ranging from 35 to 94. This supports the monophyly of subg. *Cerasus* from a mitochondrial perspective. Originally recognized as a distinct genus (Linnaeus, 1753), subg. *Cerasus* was redefined as a subgenus as phylogenetic research advanced (Gray, 1859; Wilson, 1911). To date, the interspecific relationships within subg. *Cerasus* have not been fully explored. Studies suggested that the close interspecific relationships and frequent hybridization contribute to the challenges (Hodel et al., 2021; Su et al., 2023). To further clarify the phylogenetic placement of the species, we also constructed a phylogenetic tree based on the concatenated plastid PCGs, which confirmed the monophyly of subg. *Cerasus*. Previous studies have placed *P. mahaleb* at the base of subg. *Cerasus* (Yi et al., 2024; Shen et al., 2023), a finding that was also supported by our plastid phylogeny. Comparative analysis revealed significant conflicts between the plastid and mitochondrial phylogenetic trees, highlighting the complexity of phylogenetic reconstruction within subg. *Cerasus*. These inconsistencies may arise from differences in the evolutionary rates of plastid and mitochondrial genomes, or from reticulate evolutionary events such as hybridization.

## 5 Conclusion

In this study, we assembled and conducted comparative analysis of mitogenomes of five cherry species. Our findings revealed that these mitogenomes exhibit diverse structural configurations, with limited variation in gene content and genome size across species. Notably, we observed gene loss and copy number variation in several protein-coding and tRNA genes. The repetitive sequences, particularly dispersed repeats, were prevalent and likely contributed to genomic rearrangements. Codon usage and RNA editing patterns further revealed the high conservation in five cherry mitogenomes. Phylogenetic analysis utilizing both mitochondrial and plastid PCGs substantiated the monophyly of subg. *Cerasus*, which supported the APG IV. However, phylogenetic analysis also highlighted significant incongruences between the two datasets, possibly due to differences in evolutionary rates and reticulate events like hybridization. These results underscore the complexity of mitogenome evolution in cherry species and provide valuable insights into the challenges of phylogenetic reconstruction within *Prunus*.

## Data Availability

Mitogenome sequences of the five cherry species generated in this study have been deposited in the NCBI (https://www.ncbi.nlm.nih.gov/) with accession numbers of PQ842642–PQ842646.
